# Enhancing socio-emotional communication and quality of life in young cochlear implant recipients: Perspectives from parameter-specific morphing and caricaturing

**DOI:** 10.3389/fnins.2022.956917

**Published:** 2022-08-25

**Authors:** Stefan R. Schweinberger, Celina I. von Eiff

**Affiliations:** ^1^Voice Research Unit, Friedrich Schiller University Jena, Jena, Germany; ^2^Department for General Psychology and Cognitive Neuroscience, Institute of Psychology, Friedrich Schiller University Jena, Jena, Germany; ^3^Deutsche Forschungsgemeinschaft (DFG) Research Unit Person Perception, Friedrich Schiller University Jena, Jena, Germany

**Keywords:** quality of life, children, cochlear implant, voice morphing, training

## Abstract

The use of digitally modified stimuli with enhanced diagnostic information to improve verbal communication in children with sensory or central handicaps was pioneered by Tallal and colleagues in 1996, who targeted speech comprehension in language-learning impaired children. Today, researchers are aware that successful communication cannot be reduced to linguistic information—it depends strongly on the quality of communication, including non-verbal socio-emotional communication. In children with cochlear implants (CIs), quality of life (QoL) is affected, but this can be related to the ability to recognize emotions in a voice rather than speech comprehension alone. In this manuscript, we describe a family of new methods, termed *parameter-specific facial and vocal morphing*. We propose that these provide novel perspectives for assessing sensory determinants of human communication, but also for enhancing socio-emotional communication and QoL in the context of sensory handicaps, via training with digitally enhanced, caricatured stimuli. Based on promising initial results with various target groups including people with age-related macular degeneration, people with low abilities to recognize faces, older people, and adult CI users, we discuss chances and challenges for perceptual training interventions for young CI users based on enhanced auditory stimuli, as well as perspectives for CI sound processing technology.

## Introduction

In 1996, Paula Tallal, Michael Merzenich and their colleagues published two seminal companion papers in *Science* which focused on how processing deficits in language-learning impaired (LLI) children could be ameliorated by training with acoustically modified, exaggerated speech stimuli. One paper (Merzenich et al., 1996) demonstrated that, with only 8–16 h of child-appropriate adaptive training over 20 days, LLI children improved substantially their temporal processing abilities to recognize brief sequences of both non-speech and speech stimuli. The other manuscript (Tallal et al., 1996) used speech modifications to create salient, exaggerated speech stimuli to train speech comprehension. They assessed effects of daily training over 4 weeks with temporally modified speech in which the fast (mostly consonant) parts were exaggerated relative to the slower (mostly vowel) parts. Compared with training with unmodified speech, training with temporally exaggerated speech caused far larger posttraining benefits. Importantly, benefits corresponded to about 2 years of developmental age, were achieved with only 4 weeks of training, generalized to unmodified natural speech comprehension, and were maintained at follow-up 6 weeks after training completion.

We do not know how these interventions affected quality of life (QoL) in these LLI children. But in children with hearing loss and with cochlear implants (CIs), speech recognition also has generally been treated as a benchmark for success of intervention.

At the same time, non-verbal communication skills^1^ (e.g., Frühholz and Schweinberger, 2021) have been comparatively disregarded. This seems unfortunate, because seminal work has shown only a relatively weak relationship between perceived QoL and speech recognition in CI users (Huber, 2005), and because consistent positive correlations between QoL and abilities to perceive vocal emotions were reported more recently (Schorr et al., 2009; Luo et al., 2018; von Eiff et al., 2022). These are sometimes larger than those between QoL and speech comprehension, such that the role of vocal emotions in communication can hardly be overstated (Jiam et al., 2017). In children with CIs, studies with large samples suggest that psychosocial wellbeing is tightly related to communication skills (Dammeyer, 2010). Moreover, in hearing children from low-income families, the quality of communication is a more important predictor for expressive language development in the first 3 years of life than the quantity of caregivers’ words during interaction (Hirsh-Pasek et al., 2015).

The voice—like the face—not only conveys a rich set of paralinguistic cues about a speaker’s arousal and emotions, but also more time-stable speaker characteristics including speaker identity, gender, or age (Schweinberger et al., 2014). Beyond emotions, there is now tentative evidence from adult CI users that QoL can be positively related to abilities to perceive speaker age or gender (Skuk et al., 2020), which could emphasize the importance of social information in terms of being aware who one is talking to. Nonetheless, more research is needed to understand the role of these abilities for communication success and QoL with a CI, especially in children. This can be seen in line with findings that children with a CI are particularly disadvantaged in communication and social participation, including in the school context, even when performing well on linguistic tests (Rijke et al., 2021). QoL in adults is typically regarded to depend on four pillars of physical health, psychological factors, social relationships, and environmental factors (Skevington et al., 2004). Similarly, QoL in children is adapted to four relevant domains of life relating to physical, emotional, social and school functioning (Varni et al., 2001). We define socio-emotional skills to include abilities for emotion recognition and expression, perspective taking, theory of mind, empathy, prosocial behavior, and conflict resolution (Jiam et al., 2017; Klimecki, 2019; Schurz et al., 2021). It is worth remembering that there may be bidirectional interactions between psychosocial problems and hearing performance (Shin et al., 2015), and also that socio-emotional skills appear systematically affected in children with hearing impairment more generally (for a systematic review, cf. Stevenson et al., 2015). Accordingly, many of the arguments made in this manuscript may apply to this target group as well.

## Simple morphing and parameter-specific morphing in basic science

**FIGURE 1:**
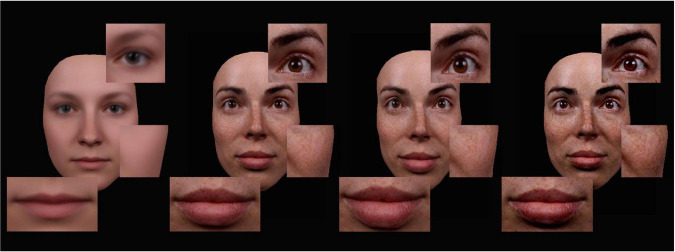
Examples for parameter-specific facial caricatures. *From left to right*: (1) An average female face, (2) a veridical individual face, (3) a shape caricature (50%) of that face (with unchanged texture information), and (4) a texture caricature (50%) of that face. Figure adapted from Limbach et al. (2022), with permission, Rightslink Ref. No. 600082258.

Soon after image morphing was invented to manipulate faces (Benson and Perrett, 1991) this triggered a real revolution to social psychophysics: Morphing suddenly allowed researchers to perform objective, quantitative, subtle and photorealistic manipulations of a social signal—regardless of whether this was identity, emotion, age, or another domain of facial variation; morphing is a general purpose technique. More than a decade later, STRAIGHT software was introduced as an analogous technology for researchers in audition (Kawahara and Matsui, 2003). With morphing, researchers can interpolate two voices (or faces), and can also create digital averages across many speakers. One key finding here is that averages are consistently perceived as more attractive than would be expected from the individual contributing faces (Langlois and Roggman, 1990), or voices (Bruckert et al., 2010). Moreover, attractiveness is negatively correlated with distinctiveness for both faces and voices (Zäske et al., 2020), and attractive faces are less memorable than unattractive ones (Wiese et al., 2014). More crucially for present purposes, a suitable average face (or voice) can serve as a reference for caricaturing: Interpolating between an average and an individual speaker can then be used to create an anti-caricature of an individual, whereas extrapolation beyond the individual can be used to create a digital caricature, in which all idiosyncratic features of speaker which deviate from the average are accentuated. Figure 1 illustrates this for faces, and shows parameter-specific caricatures of a face which were performed separately for shape and texture (note that both parameters can be combined in a full caricature).

In audition, morphing has supported investigations into neurocognitive mechanisms of voice perception with controlled stimuli, focusing on different signals such as gender (Schweinberger et al., 2008; Skuk and Schweinberger, 2014), emotions (Bestelmeyer et al., 2010, 2014; Skuk and Schweinberger, 2013; Nussbaum et al., 2022), identity (Andics et al., 2010; Latinus and Belin, 2012), or age (Zäske and Schweinberger, 2011). Here, one consistent finding has been that the perception of the same voice (e.g., one at an intermediate position on an angry-to-fearful continuum) can be influenced by previous adaptation to salient (e.g., uniformly angry) voices. Such contrastive adaptation effects were generally interpreted as evidence for norm-based coding of social signals in voices, complementing earlier analogous findings in faces. At a technical level, it should be noted that both sound and image morphing operate on computationally independent parameters which, at least in principle, can be manipulated separately (e.g., Figure 1). In image morphing, two independent parameters are *shape* (the 2D or 3D shape and metric relationships between facial features) and *texture* (skin reflectance characteristics in terms of coloration and pigmentation). Analogously, in sound morphing with TANDEM-STRAIGHT (Kawahara et al., 2008) five parameters can be manipulated separately (for technical detail, cf. Skuk and Schweinberger, 2014; Kawahara and Skuk, 2019): fundamental frequency (F0), formant frequencies (FF), spectrum level information (SL), aperiodicities (AP, including shimmer and jitter), and time (T). Note though that parameter-specific morphing (PSM) is rare—most published studies use stimuli in which all morph parameters are simply manipulated conjointly.

In the section “Clinical relevance of morphing and caricaturing: Voices” below, we propose that morphing, and PSM in particular, provides researchers with a powerful toolbox which allows, firstly, to better understand the role of auditory information for the perception of different social signals (emotion, identity, etc.) in the voice. Secondly, PSM allows to better understand and quantify individual differences in high and low performers, including in clinical conditions with sensory impairments to hearing or with central impairments (e.g., autism and phonagnosia). Finally, diagnostic information obtained with PSM can be used to develop and test tailor-made perceptual trainings, and, as a perspective, to contribute to developing tailor-made sound processing technology in CI devices. In the next section “Improving the recognition of social signals by digital caricaturing: History and perspectives”, we provide directly relevant context for this perspective.

## Improving the recognition of social signals by digital caricaturing: History and perspectives

Historically, the first computerized facial caricatures by Brennan (1985) preceded the advent of image morphing. Brennan’s work focused on computerized line drawings reminiscent of traditional hand-drawn caricatures. Thus, the first digital caricatures were entirely lacking texture (grayscale luminance or coloration) information and worked exclusively by enhancing distinctive aspects of shape (spatial positioning of salient landmarks) information. As a comment, this work may have primed researchers to assume that shape information is crucial for face recognition (e.g., Richler et al., 2009)—although it is now clear that the recognition of familiar faces from shape information alone is extremely poor (Burton et al., 2015).

A substantial proportion of studies using caricaturing to improve face recognition used shape caricatures only, leaving texture unchanged. On one hand, this seems unfortunate, because texture caricatures are more efficient than shape caricatures to improve recognition of experimentally familiarized faces (Itz et al., 2014)—a finding which generalizes to faces of different “races” relative to the observer (Zhou et al., 2021). On the other hand, the good news could be that already promising published results may actually underestimate the full potential of caricaturing.

Two recent studies used caricatured faces in a face recognition training program adapted for older adults (Limbach et al., 2018), or for young adults with poor face recognition skills (Limbach et al., 2022). Both employed 6–12 laboratory-based training sessions (ca. 1 h each) between pre- and post-training sessions, and quantified both training-induced cortical plasticity in the EEG/ERP and generalizations of training effects to other face perception tests. Training induced consistently enhanced face-sensitive ERP responses in both studies, suggesting training-induced cortical plasticity. Although behavioral benefits of this short training on face perception tests were small in magnitude, Limbach et al. (2022) showed PSM-specific training-induced improvements of training either with shape or with texture caricatures: Whereas shape-caricature training improved unfamiliar face matching, texture training elicited more marked improvements in face learning and memory. Together, this indicates that PSM caricature training is promising to improve performance in people with low face recognition skills.

## Clinical relevance of morphing and caricaturing: Faces

In vision, caricaturing has been successfully used as a general method to improve face recognition, and may work best under poor visibility conditions, in older adults (Dawel et al., 2019), or in people with lower-than-average face recognition skills (Kaufmann et al., 2013). Importantly, when initially learned as caricatures, newly learned faces can be recognized better from veridical images at test, even compared to when learned in veridical versions also (Itz et al., 2017). The Canberra group around Elinor McKone uses caricaturing to improve face recognition in people with sensory impairment due to age-related macular degeneration (AMD), and reports convincing evidence that caricaturing consistently benefits face recognition in affected individuals (Lane et al., 2018a). In parallel, the group reports evidence showing the impact of impaired face perception in AMD patients on both social interaction and QoL (Lane et al., 2018b), and identifies impaired face perception as an important contributor to lower QoL in AMD.

By implication, effective interventions to improve face perception should have potential to enhance QoL in these individuals. Note that the above-mentioned effects of caricaturing were obtained with static faces; for better transfer into everyday life, real-time facial caricaturing technology would be desirable. Although unavailable today, real-time caricaturing may approach feasibility in the foreseeable future, and may eventually be combined with bionic eyes in patients with prosthetic vision (McKone et al., 2018).

## Clinical relevance of morphing and caricaturing: Voices

To illustrate how PSM can improve assessment of voice perception, consider a recent study into the ability of adult CI users to perceive speaker gender in morphed voices in three conditions. In these conditions, acoustic information about speaker gender was preserved (a) in all STRAIGHT parameters (“full morphs”), (b) only in the F0 contour (“F0 morphs”; with all other parameters set to a non-informative intermediate level), or (c) only in vocal timbre (“timbre morphs,” which reflect a combination of FF, SL, and AP information). Although F0 and timbre both contribute to speaker gender perception in normal-hearing listeners, this study revealed that adult CI users exclusively used F0 cues to perceive speaker gender, and did not make efficient use of timbre in this task (Skuk et al., 2020). This might be thought to reflect inefficient transmission of timbre cues by a CI, but another recent study with analogous methods and participant samples seems to exclude this simple interpretation: In a task to perceive vocal emotions, adult CI users were far more efficient to use timbre, compared to F0 information (von Eiff et al., 2022). In addition, both studies revealed that these qualitative differences are seen despite large interindividual differences between CI users. This suggests that the processing of acoustic information with a CI should be considered in the context of the target social signal.

Together, PSM can help to understand sensory determinants of successful recognition of communicative signals and their impairments. But PSM might also help to devise tailor-made training programs with acoustically enhanced stimuli, for instance by exaggerating aspects of the signal that can still be processed relatively efficiently by an individual child or adult with a CI. Arguably, the prospects of such an approach depends on the potential of caricatures to improve the recognition of socio-emotional signals. Thus, we discuss relevant evidence in the next section “Discussion.”

**FIGURE 2:**
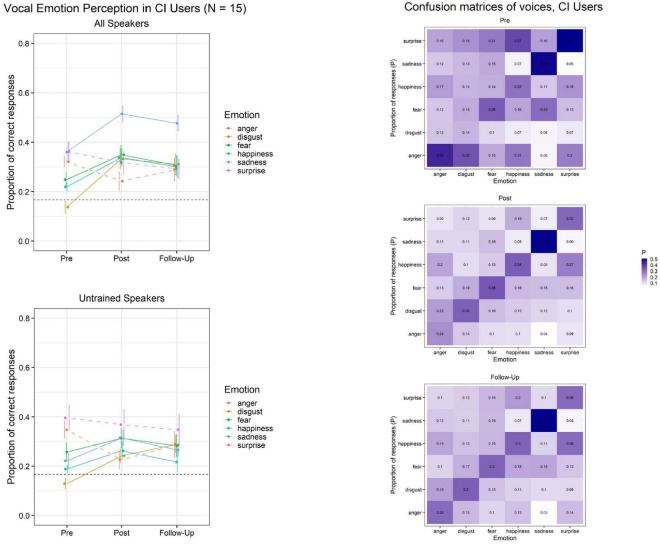
Initial outcomes from an ongoing online training (∼30 daily training sessions á 64 trials/7 min). Adult CI users (*N* = 15) were trained using caricatures of vocal emotions. Left: Lab data on vocal emotion recognition at pre-training, post-training, and follow-up. Training encompassed four emotions (disgust, fear, happiness, and sadness). Top Left: Data on utterances from all speakers. Consistent training benefits appear for trained but not untrained emotions, and are partially maintained at follow-up ∼8 weeks after training completion. Bottom Left: Data on utterances from untrained speakers only, who were never heard during training. Training benefits may generalize in attenuated magnitude across the same emotions when tested with utterances from untrained speakers. Dotted horizontal lines indicate chance levels for six response alternatives, and error bars indicate standard errors of the mean. Right: Confusion Matrices at pre-training, post-training, and follow-up. Darker hues along the diagonal (bottom left-top right) and lighter hues elsewhere correspond to better performance.

Importantly, although there is still a relative lack of research on caricatures of voices, a recent study has established that morphing of vocal emotions causes linear effects to the perception of emotional intensity and arousal, with caricatured emotions obtaining the highest ratings (Whiting et al., 2020). Together with our own groundwork, these results have encouraged us to develop an online training program which utilizes vocal caricatures of emotions to enhance vocal emotion recognition in CI users. Figure 2 illustrates initial results from this currently ongoing study, which tentatively suggest to us that caricature training indeed might be promising to improve vocal emotion recognition in CI users. We note that these findings clearly will need to be substantiated in a full study which also controls for procedural training effects; until then, they should be seen as preliminary, and interpreted with due caution.

## Discussion

Our current training program involves three (pre-training, post-training, and follow-up) lab-based sessions to optimize standardization and data quality for evaluation purposes. At the same time, accessibility and user-friendliness of the training itself should be given high priority. From a feasibility perspective, lab-based training is time-consuming, requires substantial personnel assistance, and often implicates few but long training sessions (i.e., massed rather than distributed training). On-line (or mobile) training versions, in which participants exercise at home in more frequent but shorter training sessions can facilitate transfer of acquired skills into daily life. More and shorter training session also may better exploit well-known benefits of distributed over massed learning, which seem operational for children at least from primary school ages (e.g., Ambridge et al., 2006). Trainings at home are convenient, cost-effective and accessible, particularly for children in families with financial or geographical difficulties for accessing center-based services. There is increasing proof-of-efficiency for such home-based trainings in the domain of social skills (e.g., Beaumont et al., 2021).

A key issue for contributions to this Special Research Topic is how QoL should be best assessed in children with a CI, who often receive their implant early in life. Good arguments to prefer self-reports over parent (or caregiver) reports include that there tends to be only limited agreement between these, and that young children from 5 years of age can give increasingly detailed and reliable self-ratings of QoL, provided that child-centered and age-appropriate instruments are used (Huber and Havas, 2019), such as the KINDL-R (e.g., Zaidman-Zait et al., 2017) or the PedsQL (Varni et al., 2001) and its derivates. We hypothesize that efficient trainings of vocal emotion recognition will have positive effects on several domains of pediatric QoL, particularly on emotional, social, and school functioning.

Provided the training benefits with adult CI users (Figure 2) are confirmed upon study completion, this would indicate that the potential of PSM-based caricature trainings calls for in-depth exploration. We believe that such training development and evaluation should proceed in parallel with basic research on PSM methods in voice perception. As one of the next steps, we anticipate the development and evaluation of a child-friendly version of the training targeted at children with a CI. Within the format restrictions of this article, we cannot discuss the potential of audiovisual (voice with congruent dynamic facial information) versions of a training, but given the multimodal nature of emotional communication (Young et al., 2020), audiovisual trainings may be both promising and timely: Recent research has revealed adaptive benefits from visual facial information, thus modifying earlier beliefs that effectively discouraged the use of visual stimulation during rehabilitation with a CI (Lyness et al., 2013; Anderson et al., 2017; Mushtaq et al., 2020). Of relevance, there is enormous current progress in digital speech synthesis technology (Yamagishi et al., 2012; Sisman et al., 2021). Our understanding from cross-field personal communications at the first interdisciplinary conference on voice identity (VoiceID; see Asadi et al., 2022) is that technological progress may soon enable real-time caricaturing of voices. Accordingly, this approach could inform intelligent and adaptive CI sound processors to enhance interaction quality. From a user’s viewpoint, it seems central to evaluate any evidence-based future training programs against the degree to which they cannot only improve socio-emotional communication, but also QoL in young CI recipients.

## Conclusion

Socio-emotional skills are of key importance for QoL in young CI recipients. Unfortunately, skills to perceive emotions and other non-verbal communicative signals from the voice have been neglected by previous research which, we argue, overfocused on speech recognition as the main benchmark for CI success. We show perspectives for how parameter-specific morphing and caricaturing can provide a methodological toolbox for better individual assessment and intervention in the domain of voice perception. Vision impairment and face perception are examples from a related domain for which caricaturing was already shown to improve communication and QoL; we present arguments and first results that advocate this approach for CI users. In summary, this manuscript provides perspectives both for more efficient perceptual training programs and for enhanced sound processing technologies that may benefit socio-emotional communication and QoL with a CI.

## Data availability statement

The original contributions presented in the study are included in the article/supplementary material, further inquiries can be directed to the corresponding author.

## Ethics statement

Written informed consent was obtained from the individual(s) for the publication of any potentially identifiable images or data included in this article.

## Author contributions

SRS: conceptualization, writing—initial draft, and review and editing. CIvE: conceptualization, writing—review and editing. Both authors contributed to the article and approved the submitted version.

## Footnotes

1 Please note that we do not refer to sign languages in this manuscript, but to paralinguistic social-communicative vocal and facial signals (e.g., a speaker’s emotions).
